# Establishing serum protein electrophoresis reference intervals in free-ranging barren-ground caribou (*Rangifer tarandus granti*)

**DOI:** 10.3389/fvets.2025.1717426

**Published:** 2026-01-07

**Authors:** Maris J. Daleo, Camilla Lieske, Carolyn Cray, Kimberlee B. Beckmen

**Affiliations:** 1 Division of Wildlife Conservation, Alaska Department of Fish and Game, Fairbanks, AK, United States; 2 College of Veterinary Medicine, University of Illinois Urbana-Champaign, Urbana, IL, United States; 3 Department of Pathology & Laboratory Medicine, Miller School of Medicine, Miami, FL, United States

**Keywords:** barren-ground caribou, *Rangifer tarandus granti*, reference intervals, acute phase proteins, protein electrophoresis

## Abstract

Barren-ground caribou (*Rangifer tarandus granti*) populations range in parts of Alaska, USA, and the Yukon Territory, Canada, and are culturally and nutritionally important to northern Indigenous communities and subsistence hunters. Baseline clinical metrics in free-ranging caribou can inform individual and population health, signal environmental change, and guide conservation efforts. Serum proteins, when quantified and examined using protein electrophoresis, can reflect nutritional status, metabolic issues, and organ function. However, reference intervals for these parameters are lacking in free-ranging caribou. This study established reference intervals for serum protein electrophoresis fractions in sera collected from free-ranging barren-ground caribou for the fall, spring, and summer seasons. From 1998 to 2024, 143 wild, apparently healthy breeding adults were captured across six herds in Alaska and Canada. Reference intervals were calculated for adult females in the fall (*n* = 35), summer (*n* = 34), and spring (*n* = 74), and for male and female yearlings (10–14 months; *n* = 53), following established guidelines by the American Association of Veterinary Clinical Pathology. The greatest seasonal divergence occurred between spring and fall in adult females, likely reflecting changes in pregnancy and nutrition. Protein concentrations also appeared to be useful for ruling out parasitic and bacterial infections than for detecting them; nonetheless, serum proteins provide a broad, early indication of nonspecific health disturbances. These season-specific reference intervals offer a baseline for assessing the health of free-ranging caribou herds.

## 1 Introduction

The acute phase response and the humoral immune response both alter hepatic protein synthesis and, consequently, the composition of serum proteins. These changes can provide early, nonspecific indicators of stressors affecting individual and population-level wildlife health (1, 2). Serum protein electrophoresis (SPE) has been demonstrated as a useful tool for quantifying these shifts by measuring albumin and separating globulins into alpha-1 (α1), alpha-2 (α2), beta (β), and gamma (γ) fractions, along with the albumin-to-globulin (A:G) ratio. In wildlife medicine, SPE can aid in early disease detection by revealing coordinated inflammatory patterns that may precede leukogram abnormalities and conventional serum biochemistry changes (3, 4). Because rapid immune-driven changes are reflected in these fractions, SPE provides an integrative, pattern-based index of host response (3, 5–7).

The four globulin fractions have different sources and functions and can be used to monitor various health conditions (9). Alpha-1 globulins are proteins produced in the liver involved in the acute phase response, hormone transport, and protease inhibition (10). Their levels typically increase during inflammation, infection, or trauma, primarily due to increased levels of IL-6 and TNF-α (11). Low levels of alpha-1 globulins can suggest α1 antitrypsin deficiency, liver and/or kidney disease, or malnutrition (2, 3, 10, 11). Alpha-2 globulins bind free hemoglobin, transport copper, and inhibit proteases (1, 10). Alpha-2 globulin concentrations can increase with inflammation, pneumonia, or sepsis and may decrease with hemolysis, kidney disease, or copper imbalances (9). For example, haptoglobin, an alpha-2 globulin, is an acute phase protein commonly used as an inflammatory marker in cattle and other ruminants, including reindeer and caribou (2, 12–15). Beta globulins contribute to iron transport, complement activation, and support for the major histocompatibility complex class I (10). Elevated beta-globulins can indicate infection, anemia, or hematologic disorders, while decreased levels may reflect chronic illness or malnutrition (10, 16). Gamma globulins are antibodies produced by plasma cells and are essential for the humoral immune response (17). Their levels typically rise in chronic infections or autoimmune disease and are reduced in immunodeficiencies or pregnancy (10, 17). Baseline parameters of these proteins can help distinguish between local and large-scale stressors affecting free-ranging wildlife, particularly those that are culturally and environmentally crucial, such as cervids.

Barren-ground caribou (*Rangifer tarandus granti*) populations are found in parts of Alaska, USA, and the Yukon Territory, Canada (18). These caribou are migratory and are managed by herd based on their traditional core calving grounds, to which females return annually (19). Barren-ground caribou are important to northern subsistence hunters, especially in indigenous cultures and communities (18). For this reason, wildlife management agencies have developed comprehensive management plans for numerous caribou herds to establish long-term conservation objectives and preserve their traditional uses (18, 20–22). Wildlife managers and researchers have relied on telemetry, aerial surveys, and serology from blood samples obtained during live-capture release operations, supplemented with hunter-harvest monitoring, to evaluate herd health, identify disease trends, and describe the overall health of the caribou population (21, 23–28).

Despite studies that monitor and quantify the acute phase response for detecting health abnormalities, reference ranges for specific serum proteins from SPE in healthy caribou have not been published (5–7, 29). Only a few serum protein reference intervals have been established in captive cervids, including reindeer (*Rangifer tarandus tarandus*) and white-tailed deer (*Odocoileus virginianus*), and only serum amyloid A and haptoglobin have been studied in free-ranging caribou (5–8). Protein electrophoresis can be a valuable tool in herd health monitoring, enabling the ante-mortem, non-specific detection of subclinical disease and inflammation. Numerous studies have reported alterations in serum protein levels in both diseased and non-diseased individuals (1–3, 11, 14, 29). Beyond individual animal health, serum proteins are increasingly recognized as valuable biomarkers for assessing broader ecosystem health (1). Therefore, the purpose of this study is to determine reference intervals for barren-ground caribou albumin and protein fractions based on serum protein electrophoresis results across three seasons for two age classes and to assess the utility of serum globulins in identifying caribou populations with health concerns to be further investigated.

## 2 Materials and methods

Protein electrophoresis was performed on archived serum samples collected on 825 adult and yearling free-ranging caribou during live-capture release studies by Alaska Department of Fish and Game biologists between 1998 and 2024. Herds of origin included the Central Arctic Herd, Fortymile Caribou Herd, Nelchina Caribou Herd, Teshekpuk Caribou Herd, Western Arctic Caribou Herd, and Mulchatna Caribou Herd. Caribou were captured using chemical immobilization or physical restraint, depending on season and herd. The season of capture was reported as fall (August-October), spring (March-May), and summer (June-July). Age was determined based on animal size and categorized as adult (>3 years) or yearling (10–14 months). Blood was collected from the jugular or cephalic vein into evacuated tubes (BD Vacutainer^®^ SST™ Tubes, Becton, Dickinson and Company, Franklin Lakes, New Jersey, USA). Tubes were centrifuged at 1,000–2,000 x g for 10 min, and the harvested serum was transferred to cryovials and frozen in a dry nitrogen shipper for transport. The sera were stored in cryovials at −80 °C until shipment on dry ice to the Acute Phase Protein Laboratory at the University of Miami for protein electrophoresis analysis.

Serum protein electrophoresis was conducted using a SPIFE 3000 system (Helena Laboratories, Beaumont, Texas, USA) and split beta gels. Total protein was determined by a non-temperature-compensated refractometer. Five fractions were quantified by densitometry: albumin, alpha-1 globulins, alpha-2 globulins, beta globulins, and gamma globulins. The albumin-to-globulin ratio (A:G) was calculated as the albumin concentration divided by the total globulins (3). Reference intervals for each season were calculated separately to account for seasonal variation in serum protein fraction levels using adult females to investigate how proteins change during pregnancy. Serum protein fraction intervals were also calculated for male and female yearlings, which were only captured in the summer and spring. Only the samples from the initial capture were used for recaptured individuals in this analysis to determine reference intervals. Caribou sera were excluded from the reference interval calculation if there were any reports of external signs of clinical illness, known mortality within 30 days of capture, or hemolysis in the serum sample. Additional serological tests were also performed to exclude individuals with antibodies indicative of exposure to *Brucella suis* biovar 4, *Toxoplasma gondii, Neospora caninum*, and *Erysipelothrix rhusiopathiae*.

To identify potential outliers in serum protein values for each season, we applied Tukey's Interquartile Range method, removing outliers with values more than one and a half interquartile ranges beyond the first or third quartile, as per the American Society for Veterinary Clinical Pathology (ASVCP) guidelines (30). In guideline concordance, 90% confidence intervals were calculated for each protein analyte using a bootstrapping analysis, which involved resampling the observed data with replacement and computing quantiles across 1,000 bootstrap replicates (30). Distribution was classified as Gaussian if normally distributed, based on a Shapiro-Wilk test p-value (*p* < 0.05), or non-Gaussian otherwise, following ASVCP guidelines (30, 31).

To assess the diagnostic utility of protein biomarkers in detecting pathogen exposure, the sensitivity and specificity were calculated for each protein in relation to bacterial (*Brucella suis* biovar 4 and *Erysipelothrix rhusiopathia*) and parasitic (*Toxoplasma gondii* and *Neospora caninum*) exposure. The sensitivity for each protein fraction and pathogen group was computed by considering the number of pathogen-seropositive individuals outside the reference interval (true positives) and the number of pathogen-negative individuals with values outside the reference interval (false positives). We also calculated the ability of each protein to distinguish pathogen-negative individuals (specificity) by comparing the proportion of pathogen-negative individuals outside (false positives) and within (true negatives) the reference interval.

## 3 Results

**Figure 1:**
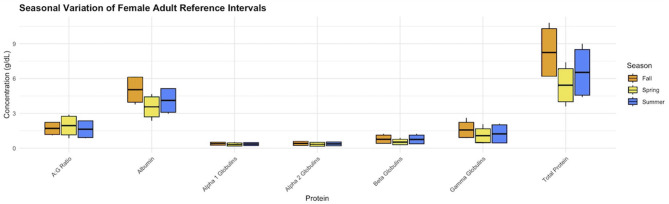
Seasonal changes in serum protein reference intervals for female adult barren-ground caribou (*Rangifer tarandus granti*). Shaded boxes represent the reference intervals for each protein during that season, and each box's midline represents the reference interval's midpoint. Lines stemming from each box represent the 90% confidence intervals for the respective reference range.

Seventy-six animals in the dataset were recaptured, and only the first capture encounter samples for these individuals were considered for inclusion. There were 23 individuals seropositive for *Erysipelothrix rhusiopathiae* exposure, 141 animals seropositive for *Neospora caninum* exposure, 95 animals seropositive for *Toxoplasma gondii* exposure, and 32 animals seropositive for *Brucella suis* biovar 4 exposure. There were 215 animals with at least one serum protein outlier removed from the dataset. Additionally, 103 individuals were removed due to external signs of illness, mortality, or hemolysis. After excluding these 609 individuals, only 20 adult males were removed from the dataset. Due to the low sample size of individuals meeting the inclusion criteria, reference intervals were created for adult females and yearlings of both male and female sex. Reference intervals were established for each protein during the fall (Table 1), summer (Table 2), and spring (Table 3) capture seasons using 143 adult females (Figure 1). Additional intervals were calculated for 53 male and female yearlings (Table 4).

**Table 1:** Reference intervals (RI) for serum proteins (g/dL) in 35 adult female barren-ground caribou (*Rangifer tarandus granti*), including the mean, standard deviation (SD), median, minimum (min), and maximum (max) for each analyte in the fall (August-October).

***N***	**Protein**	**Mean**	**SD**	**Median**	**Min**	**Max**	**RI**	**LRI 90% CI**	**URI 90% CI**	**D**	***p*-value**
35	A:G Ratio	1.57	0.29	1.52	1.11	2.24	1.18–2.23	1.11–1.95	1.95–2.24	G	0.46
	Albumin (g/dL)	4.64	0.58	4.53	3.77	6.13	3.96–6.11	3.77–4.09	5.40–6.13	G	0.88
	Alpha 1 Globulins (g/dL)	0.36	0.08	0.32	0.24	0.54	0.26–0.51	0.24–0.29	0.48–0.54	G	0.77
	Alpha 2 Globulins (g/dL)	0.40	0.11	0.39	0.23	0.61	0.24–0.58	0.23–0.27	0.57–0.61	G	0.15
	Beta Globulins (g/dL)	0.74	0.18	0.75	0.39	1.24	0.42–1.13	0.39–0.56	0.94–1.24	G	0.59
	Gamma Globulins (g/dL)	1.58	0.41	1.57	0.87	2.62	0.92–2.23	0.87–1.05	2.02–2.62	G	0.19
	Total Protein (g/dL)	7.75	1.14	7.60	6.20	10.80	6.20–10.3	6.20–6.60	9.20–10.80	G	0.77

Lower (LRI) and upper reference intervals (URI) are reported with 90% confidence intervals (CI). Distribution (D) is classified as Gaussian (G) if normally distributed, based on the Shapiro-Wilk test *p*-value. Caribou were captured in Alaska, USA, during the fall (August–October).

**Table 2:** Reference intervals (RI) for serum proteins (g/dL) in 34 adult female barren-ground caribou (*Rangifer tarandus granti*), including the mean, standard deviation (SD), median, minimum (min), and maximum (max) for each analyte in the summer (June-July).

***N***	**Protein**	**Mean**	**SD**	**Median**	**Min**	**Max**	**RI**	**LRI 90% CI**	**URI 90% CI**	**D**	***p*-value**
34	A:G Ratio	1.65	0.39	1.64	0.86	2.38	0.91–2.36	0.86–1.22	2.17–2.38	G	0.85
	Albumin (g/dL)	3.82	0.62	3.60	2.97	5.17	3.09–5.14	2.97–3.23	4.82–5.17	nG	0.002
	Alpha 1 Globulins (g/dL)	0.34	0.08	0.32	0.21	0.51	0.23–0.49	0.21–0.26	0.45–0.51	nG	0.04
	Alpha 2 Globulins (g/dL)	0.33	0.10	0.29	0.18	0.55	0.21–0.54	0.18–0.24	0.44–0.55	nG	0.01
	Beta Globulins (g/dL)	0.65	0.21	0.66	0.35	1.24	0.38–1.13	0.35–0.42	0.87–1.24	nG	0.04
	Gamma Globulins (g/dL)	1.15	0.44	1.12	0.44	2.11	0.45–2.02	0.44–0.64	1.65–2.11	G	0.38
	Total Protein	6.3	1.14	6.00	4.4	9.00	4.56–8.50	4.40–5.10	7.80–9.00	G	0.28

Lower (LRI) and upper reference intervals (URI) are reported with 90% confidence intervals (CI). Distribution (D) is classified as Gaussian (G) if normally distributed, based on the Shapiro-Wilk test *p*-value, or non-Gaussian (nG) otherwise, following ASVCP guidelines. Caribou were captured in Alaska, USA, during the summer (June–July).

**Table 3:** Reference intervals (RI) for serum proteins (g/dL) in 74 adult female barren-ground caribou (*Rangifer tarandus granti*), including the mean, standard deviation (SD), median, minimum (min), and maximum (max) for each analyte in the spring (March-May).

***N***	**Protein**	**Mean**	**SD**	**Median**	**Min**	**Max**	**RI**	**LRI 90% CI**	**URI 90% CI**	**D**	***p*-value**
74	A:G Ratio	1.95	0.48	1.94	0.86	2.89	1.15–2.74	0.86–1.28	2.68–2.89	G	0.37
	Albumin (g/dL)	3.45	0.47	3.48	2.36	4.66	2.70–4.43	2.36–2.81	4.12–4.66	G	0.67
	Alpha 1 Globulins (g/dL)	0.29	0.08	0.27	0.18	0.53	0.19–0.45	0.18–0.21	0.43–0.53	nG	0.00
	Alpha 2 Globulins (g/dL)	0.28	0.09	0.27	0.15	0.52	0.17–0.50	0.15–0.18	0.44–0.52	nG	0.00
	Beta Globulins (g/dL)	0.47	0.11	0.47	0.28	0.89	0.30–0.76	0.28–0.33	0.61–0.89	nG	0.00
	Gamma Globulins (g/dL)	0.85	0.33	0.74	0.41	2.07	0.47–1.68	0.41–0.51	1.37–2.07	nG	0.03
	Total Protein	5.36	0.76	5.20	3.60	7.40	4.00–6.85	3.60–4.40	6.60–7.40	G	0.26

Caribou were captured in Alaska, USA, during the spring (March–May).

**Table 4:** Serum protein (g/dL) reference intervals (RI) for yearling (10–14 months) male and female barren-ground caribou (*Rangifer tarandus granti*), including the mean, standard deviation (SD), median, minimum (min), and maximum (max) for each analyte in the spring (March–May) and summer (June and July).

***N***	**Protein**	**Mean**	**SD**	**Median**	**Min**	**Max**	**RI**	**LRI 90%CI**	**URI 90% CI**	**D**	***p*-value**
53	A:G Ratio	1.38	0.27	1.39	0.84	1.90	0.89–1.88	0.84–1.00	1.70–1.90	G	0.45
	Albumin (g/dL)	3.56	0.45	3.50	2.48	4.54	2.90–4.39	2.48–3.00	4.20–4.54	G	0.56
	Alpha-1 Globulins (g/dL)	0.39	0.05	0.39	0.26	0.54	0.31–0.51	0.26–0.32	0.46–0.54	G	0.36
	Alpha-2 Globulins (g/dL)	0.38	0.08	0.36	0.25	0.58	0.25–0.53	0.24–0.27	0.50–0.58	nG	0.03
	Beta Globulins (g/dL)	0.70	0.15	0.67	0.47	1.15	0.48–0.96	0.47–0.51	0.90–1.15	nG	0.03
	Gamma Globulins (g/dL)	1.22	0.38	1.17	0.57	2.13	0.70–2.08	0.57–0.74	1.74–2.13	G	0.11
	Total Protein (g/dL)	6.26	0.76	6.20	4.80	8.0	5.00–7.58	4.80–5.20	7.40–8.00	G	0.59

Lower (LRI) and upper (URI) reference limits are reported with 90% confidence intervals (CI). Distribution (D) is classified as Gaussian (G) if normally distributed, based on the Shapiro-Wilk test *p*-value, or non-Gaussian (nG) otherwise, following ASVCP guidelines.

Yearling caribou were captured in Alaska, USA, during the spring (March–May) and summer (June and July).

The sensitivity for detecting proteins associated with parasitic infections was low, ranging from 5.3% (gamma globulins) to 11.2% (albumin). Specificity for detecting parasitic infections was relatively high, ranging from 83.7% (gamma globulins) to 90% (beta globulins), suggesting that protein abnormalities are not reliable indicators of parasite exposure. However, reference protein values may have some utility in excluding parasitic infections. The sensitivity was slightly higher for detecting exposure to bacterial pathogens, with total protein (25.5%), albumin (23.6%), and alpha-1 globulins (18.2%) exhibiting the highest sensitivities. Similarly to parasite infections, the specificity for detecting bacterial pathogen exposure was high amongst all proteins, ranging from 86.5% (gamma globulins) to 91.7% (beta globulins).

## 4 Discussion

This study presents reference intervals for serum proteins in free-ranging barren-ground caribou. Our reference intervals are comparable to those published for reindeer, white-tailed deer, and cattle (5–7). Furthermore, our reference intervals for caribou exhibited lower alpha-1 and alpha-2 values than all other species (5–7). The caribou values also showed higher beta and gamma, and globulin levels were lower than those of free-ranging Norwegian reindeer, although the reference range calculation included only 21 females within a captive facility during a translocation program (6). Extreme cold and nutritional variability can impact protein metabolism and alter protein reference intervals compared to temperate species, such as cattle or white-tailed deer (20, 26, 27). Our reference intervals may also differ from those of other species due to field conditions, stress during capture, and unreported serum hemolysis, which can all impact protein profiles differently in caribou compared to captive or domestic species. This variation in protein values between ungulate species underscores the importance of developing species-specific reference intervals for accurate health assessments in free-ranging populations.

The greatest seasonal variation in reference intervals was observed between the fall and spring capture seasons, likely due to the stress of pregnancy. The largest change in reference interval was observed in total serum proteins, with the highest range occurring in the fall and the lowest interval in the spring for adult females. Previous studies have shown that pregnancy was associated with a small, significant decrease in total protein during the later gestational stages in May (32). The intervals in our study for total serum protein vary significantly between seasons, but the purpose of this analysis was to characterize general reference ranges for free-ranging caribou herds throughout the year, which may fluctuate due to reproductive or nutritional changes. Similarly, previous caribou protein studies observed a decrease in albumin in the spring, which we also observed in our reference intervals (32). The seasonal patterns in blood total protein and albumin concentrations, characterized by higher concentrations in autumn, may be due to changes in nutrition between summer and winter (32, 33). Nutrition, age, and pregnancy can all affect the variation in serum proteins, which is typically characterized by lower concentrations in pregnant animals (9, 32, 33).

Across all pathogen groups, no single protein performed well as a sensitive independent diagnostic for bacterial or parasitic exposure. Sensitivities across proteins were uniformly low, likely reflecting the nonspecific nature of the acute phase response, which can be triggered by infection, trauma, or other stressors (2, 11). The low sensitivities indicate that alterations in serum protein levels are not associated with exposure to the four pathogens investigated in this study. However, the relatively high specificity across all proteins implies that protein levels may be more effective at ruling out infection than detecting it. Because the ELISA assays in this study measure seropositivity, which reflects prior exposure rather than active infection, serum protein values are likely to return to baseline after inflammation resolves. For example, elevated serum protein levels in a seropositive animal could suggest ongoing inflammation, whereas protein values closer to baseline are more consistent with past exposure or resolved infection. Protein electrophoresis quantifies albumin and the protein fractions, capturing coordinated shifts typical of early inflammation—changes that can precede leukogram abnormalities and clinical signs (1, 11). Therefore, while single serum protein fractions may not be sensitive for detecting pathogen-specific exposure, measuring the protein fractions provides a broader, pattern-based signal that is often more sensitive to early, nonspecific health perturbations and can aid in triaging animals for more targeted diagnostics (11). Identifying abnormal serum proteins can provide a basis for an inflammatory etiology, which can aid in developing early diagnosis and treatment, even if not pathogen-specific (2, 3, 11).

While efforts were made to include only healthy animals in the reference groups, there are limitations in evaluating health indices in wild populations compared to captive populations. Consequently, some outliers may have exhibited subclinical inflammation, despite having elevated protein values. We excluded individuals from our calculations using pathogen exposure to four pathogens, external signs of illness, and known mortality. Although some individuals with known mortality or clinical signs were excluded from this analysis, there may have been individuals with clinical signs that were not recorded in the dataset, as physical exams could have varied among individuals performing them. Additionally, serology provides information on retrospective exposure, and a seropositive animal does not necessarily indicate an unhealthy individual; however, we chose to exclude individuals who were seropositive to any pathogen. We also removed individuals with any serum protein outlier to eliminate individuals who may have been harboring subclinical disease. Although we used standard methods only to include apparently healthy individuals, there may have been underlying infections within our population that we were unable to detect, survey, or visualize during physical examinations.

After eliminating individuals with known mortality, pathogen exposure, clinical signs, and serum protein outliers, only 20 adult males remained across all three seasons. Due to the limitation in sample size, we chose not to include adult males in this analysis. The goal of this analysis was to establish reference intervals for adult females, which will aid in population health surveillance. To make the reference intervals more applicable, we chose to include reference intervals for yearlings, which are too young to experience pregnancy, so including males and females together made sense to incorporate a larger sample size. However, the applicability of these intervals may be limited by the exclusion of adult males, and future studies with large sample sets should aim to produce reference intervals for adult male caribou.

Because the data in our analysis span 26 years, there is potential for confounding effects resulting from long-term variations in analytical, environmental, or sampling integrity. The goal of this analysis was to incorporate seasonal variations in serum protein levels to account for pregnancy during the spring. However, variations may occur due to environmental changes that have affected the expression of proteins not quantified in this analysis. Furthermore, while protocols attempt to account for consistency between sampling years, this analysis is limited by the potential confounding effects from variations in environmental and sample integrity.

Overall, the results of the current study provide a starting point for clinically applicable reference intervals for serum total protein and protein fractions in free-ranging barren-ground caribou. These reference intervals are crucial for understanding changes in herd health over time, particularly as herds encounter rapid environmental shifts associated with a changing climate. Establishing reference intervals creates a baseline for long-term health monitoring and offers a baseline for identifying nonspecific health perturbations at the population level. Although our reference intervals show promise in identifying non-specific health issues in caribou herds, further studies are required to comprehend the intricate roles serum proteins play within free-ranging wildlife, particularly in a herd setting, to better identify herds that may require additional conservation efforts. This work is crucial for wildlife conservation and management in Alaska and Canada, as it enables the monitoring of cervid health in the face of stressors such as infectious diseases and environmental changes. Furthermore, the clinical nature of these baseline values will provide a valuable basis for assessing free-ranging caribou during herd surveys.

## Data Availability

The datasets presented in this study can be found in an online repository: https://doi.org/10.5281/zenodo.17236982. Individual-level data are owned by the Alaska Department of Fish and Game and anonymized data are available from the custodian upon reasonable request, subject to a data use agreement.
